# Ants learn fast and do not forget: associative olfactory learning, memory and extinction in *Formica fusca*

**DOI:** 10.1098/rsos.190778

**Published:** 2019-06-19

**Authors:** Baptiste Piqueret, Jean-Christophe Sandoz, Patrizia d'Ettorre

**Affiliations:** 1 Laboratory of Experimental and Comparative Ethology (LEEC), University of Paris 13, Sorbonne Paris Cité, 93430 Villetaneuse, France; 2 Evolution, Genomes, Behaviour and Ecology, CNRS, Université Paris-Sud, IRD, Université Paris-Saclay, 91190 Gif-sur-Yvette, France

**Keywords:** conditioning, learning and memory, olfaction, protein synthesis inhibitor, social insects

## Abstract

Learning is a widespread phenomenon that allows behavioural flexibility when individuals face new situations. However, learned information may lose its value over time. If such a memory endures, it can be deleterious to individuals. The process of extinction allows memory updating when the initial information is not relevant anymore. Extinction is widespread among animals, including humans. We investigated associative appetitive learning in an ant species that is widely distributed in the Northern Hemisphere, *Formica fusca*. We studied acquisition and memory between 1 h and one week after conditioning, as well as the extinction process. Ants learn very rapidly, their memory lasts up to 3 days, decreases slowly over time and is highly resistant to extinction, even after a single conditioning trial. Using a pharmacological approach, we show that this single-trial memory critically depends on protein synthesis (long-term memory). These results indicate that individual ant workers of *F. fusca* show remarkable learning and memory performances. Intriguingly, they also show a strong resistance to updating learned associations. Resistance to extinction may be advantageous when the environment is stochastic and individuals need to switch often from one learned task to another.

## 1. Background

Behavioural flexibility offers significant fitness advantages, especially in environments where resource distribution or threats are characterized by stochasticity. One way to achieve this flexibility is via learning, defined as a change in behaviour occurring as a result of experience. Many learned behaviours can be further modified to suit changing environmental conditions. The ability to learn and memorize allows animals to respond to environmental stimuli in an adaptive way, for instance, by either ignoring them or by giving them a specific value, positive or negative. This helps in predicting the environment when facing new but similar situations [1].

Storing information is costly; therefore, only essential pieces of information should remain available for the individual. For instance, with time, a stimulus which used to predict a certain resource in the environment (e.g. the presence of food) might lose its significance and be no longer associated with the resource. It is beneficial to learn rapidly that such a stimulus is not reliable anymore. Extinction is the process in which a conditioned response gradually decreases through repeated experience with the stimulus in the absence of its outcome. Extinction generally involves the formation of a new inhibitory memory rather than the destruction of the previous memory [2]. Knowledge about the extinction process has important clinical applications, for instance, for the treatment of drugs addiction and abnormal fear of a past event (e.g. war trauma) in humans [3].

The extinction phenomenon was first described by Pavlov in 1927 in experiments with dogs using classical conditioning, the association of an unconditioned stimulus (US, for example a reward) with an initially neutral stimulus that becomes a conditioned stimulus (CS) producing the response in the absence of the US. After a successful conditioning (CS–US association), Pavlov observed the conditioned responses stopped after a few unrewarded CS presentations, leading to the extinction of the conditioned behaviour. Extinction does not erase the old memory. It is rather a new learning (creation of a CS–no US association). Therefore, two memories coexist. When time passes after successful extinction, the original behaviour may reappear (called spontaneous recovery or relapse), through a decay of the extinction memory [2]. Associative learning and extinction are widespread in the animal kingdom and have been intensively studied in several vertebrate species such as mice [4] or zebrafish [5] and also in invertebrates species such as snails [6], crabs [7] or nematodes [8]. Among invertebrates, insects like fruit flies became key model species for learning and memory [9–11]. Insects are well suited for laboratory studies because they are relatively easy to keep, they have short reproductive cycles and offer easy access to brain structures (e.g. crickets, [12]). Learning and extinction have also been investigated in social insects, including bumblebees [13,14] and honeybees [2,15]. Among social insects, ants are the most diverse group with more than 14 000 described species, which represent up to 25% of the total animal biomass on Earth [16]. Visual learning in ants has been intensively studied, also at the individual level, in the context of spatial orientation and navigation [17–19]. Individual olfactory learning has been less investigated [20–27]. Carpenter ants are very efficient in discriminating between different odorants [24,26] and even between different concentrations of the same compound [27]. Recently, workers of *Lasius niger* were shown to be able to learn odour–reward associations after only one training trial, while more trials were required when using spatial cues instead of odours [23]. However, in this study, the dynamics of memory formation was not investigated. We know that individual ants can form long-term olfactory memories after six CS–US presentations [22], but whether fewer conditioning trials lead to long-term memory (LTM) is unclear. Furthermore, data about extinction of olfactory learned associations are very scarce in ants [28].

In the present work, we present the results of a laboratory study on individual associative olfactory learning, memory and extinction in the ant *Formica fusca*. Among ants, the genus *Formica* was described as one of the most advanced from a cognitive point of view (especially concerning communication and learning) [29]. *Formica fusca* is widely distributed and lives in a variety of environments with a large range of temperatures, resources, predators and competitors. Colonies are populous (hundreds of individuals) and grow well in laboratory conditions. We investigated the acquisition performance of individual ants by changing the number of conditioning trials (from one to six). We tested ants' memory abilities by subjecting them to a memory test between 1 h and one week after training. We then categorized the memory using a pharmacological approach by administrating a protein synthesis inhibitor. Finally, we studied the extinction phenomenon in individual ants, by measuring their behaviour after unrewarded presentations of the CS.

## 2. Material and methods

### 2.1. Insects and origin of colonies

*Formica fusca* is a relatively common ant species found in the Northern Hemisphere. This species can be monogynous or polygynous and colonies may contain several hundred individuals [30]. Nine queenright colonies were collected in the forest of Ermenonville (France, 49°09′51.5″ N, 2°36′49.2″ E) in September 2013 (*n* = 5) and 2017 (*n* = 4) and kept under laboratory conditions (25 ± 2°C, 50 ± 10% relative humidity, 12 h/12 h: day/night). Tested ants were foragers and were coloured on the abdomen or thorax using oil-based paint. Each ant was used only once in the conditioning and testing procedure.

### 2.2. Odorant stimuli

Hexanal and 1-octanol (Sigma Aldrich, respectively, USA and Germany, purity greater than 99%) were used as conditioned stimuli. These compounds are found in floral emissions [31] and therefore may be ecologically relevant for *F. fusca* ants who feed on extra-floral nectar. Ants did not show a spontaneous preference for any odorant (details in electronic supplementary material).

### 2.3. Experimental protocol

#### 2.3.1. Acquisition

**Figure 1.**
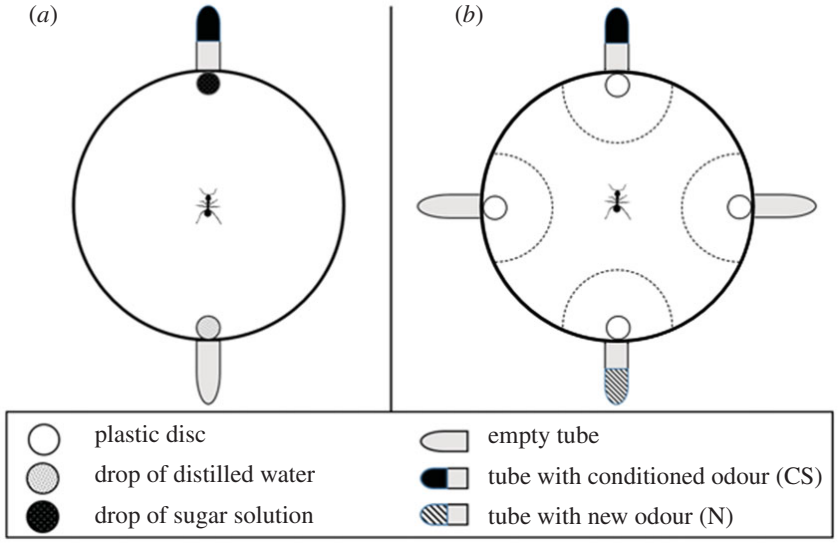
Schema of the experimental device (the arena). During conditioning, the set-up on the left was used (*a*). During memory test and extinction trials, the set-up on the right was used (*b*). The time spent by the ant in the areas around the discs was recorded during 2 min. Each area measured 35.5 cm^2^ (dashed lines). The orientation of the arena in the experimental room was changed between trials, so that ants could not learn spatial cues.

Our protocol is a modified version of that used by Bos *et al*. [21] to study perceptual similarity among cuticular hydrocarbons. In absolute olfactory conditioning, a single initially neutral odorant (CS) is associated with a reward (US). For the conditioning trials, the ant was placed in the centre of a circular arena (Ø = 12 cm, height = 3.5 cm) with clean filter paper at the bottom. The arena had two holes in the wall facing each other. Eppendorf tubes were inserted into the holes with their openings towards the centre of the arena. The tube presenting the CS contained a piece of filter paper (1 cm^2^) soaked with 1 µl of hexanal or 1-octanol, while the other tube contained a clean piece of filter paper (this tube without odour represents the control for visual and tactile cues). On each side, a cotton plug allowed passive diffusion of the odorant when present but prevented the ants from entering in direct contact with the filter papers. Small plastic discs (Ø = 6 mm) placed at 1 cm in front of each tube received 1 µl of sugar solution (30% w/w) on the CS side and 1 µl of distilled water on the other side (figure 1*a*). Due to the presence of these two drops of liquid, the two stimulus sides were visually indistinguishable. The time needed by the ant to find the sugar solution (US) was recorded during each conditioning trial. The ant was allowed to drink the drop of sugar solution and was then returned to the colony, where the ant could perform trophallaxis. During the trophallaxis, ants exchange the sugar solution drunk during the conditioning trial with nest-mates. Without trophallaxis, the crop of tested ants would be full in only a few conditioning trials and the ants would not be motivated to find more food. Trophallaxis thus ensures high and stable motivation for the tested ants. Tested ants were left for about 3 min in the colony (inter-trial interval, 186 ± 18 s, mean ± standard deviation), during which they terminated trophallaxis and came back to the foraging arena. During this interval, the filter paper at the bottom of the arena and the plastic discs were replaced with clean ones to avoid the use of any possible chemical cue left by the ant. The orientation of the arena and the position of the experimenter were also modified between trials to limit the possible use of visual or other spatial cues. Three independent groups of ants were subjected to one, three or six conditioning trials (electronic supplementary material, figure S1).

#### 2.3.2 Memory tests

For the unrewarded memory tests, we used an arena with four holes in the wall (instead of two), which were connected to four Eppendorf tubes. Four empty plastic discs were placed in front of them. There were two tubes with odorants (the CS and the novel odour, N, never encountered during conditioning) and two tubes without odorants (figure 1*b*). For ants conditioned with hexanal, 1-octanol was used as N, and conversely, for ants conditioned to 1-octanol, hexanal was N. A circular area was drawn around each tube which allowed us to record the time spent by the ant in the vicinity of each odorant for 2 min with the software Ethoc (v. 1.2, CRCA, Toulouse, France). Independent groups of ants were subjected to the memory test 1, 24, 72 or 168 h after conditioning. Each ant underwent only one memory test.

#### 2.3.3. Extinction

The extinction protocol was performed with the same arena as the memory test and started 1 h after the end of the conditioning. Each extinction trial was similar to the unrewarded memory test: the ant had the choice between CS and N for 2 min. All ants underwent at least six extinction trials. In the case where we tested the resistance to extinction after one conditioning trial, a subgroup of ants underwent six additional extinction trials, for a total of 12 extinction trials. Between two trials, each ant was returned to the colony for 3 min (inter-trial interval). During this time, the filter paper and plastics discs of the arena were replaced with new ones. Spontaneous recovery was tested 24 h after the end of the extinction protocol, by performing a last unrewarded test [32]. After this test, we offered each ant a drop of sugar solution to check whether they were motivated to feed. All ants (*N* = 88) but one drank the sugar solution. The one that did not was discarded from the analyses.

#### 2.3.4. Pharmacological treatment

Memory is divided in different categories depending on its duration and the molecular cascades it involves. For example, a long-lasting memory involving de novo protein synthesis will be qualified as LTM [11,33]. To test if ants’ olfactory memory depends on protein synthesis, additional groups of ants were given cycloheximide, a protein synthesis (translation) inhibitor (CHX, Sigma Aldrich, USA, purity greater than 99%). To prevent the drug from spreading in the colony, we created subcolonies consisting of groups of 40 ants in a nest-box with five or six larvae. A maximum of 10 of these 40 ants were tested. Each experimental ant was individually confined in a small cylinder placed inside the subcolony and received either 1 µl of sugar solution (30% w/w) containing 1 µg of CHX (treatment) or 1 µl of sugar solution (control), similarly to Guerrieri *et al.* [22]. After 2 h, the ant was released, allowing interaction with nest-mates. One hour later, therefore 3 h after receiving the treatment, the experimental ant was subjected to one conditioning trial and was then placed back into its subcolony until the memory test. This memory test was performed either 1 or 72 h after the end of conditioning. We verified that CHX did not affect ants' health (electronic supplementary material, table S4).

In total, for all experiments, 496 individual ants were conditioned, of which 467 (94%) underwent a memory test or an extinction protocol. Twenty-nine ants were excluded because they took too long to find the sugar solution during conditioning (more than 10 min for the first trial or more than 2 min for the following ones), they did not drink the CHX or control solution, or they died between conditioning and test procedures.

### 2.4. Statistics

Data were analysed using R software (v. 3.5.2, R Core Team, 2018 [34]). Significance was fixed at *α* = 5%. All data were analysed using linear mixed models (LMM, package ‘lme4’, [35]), details in electronic supplementary material. Post hoc differences were observed by using LMMs with reduced dataset and the *α*-level was adjusted using the Holm–Bonferroni correction [36].

#### 2.4.1. Acquisition

We analysed the effects of two independent (predictor) variables: *conditioning odorant* (factor with two levels, hexanal or 1-octanol) and the number of conditioning trials (continuous variable up to 6, named *trials*) on the dependent variable *time* (continuous variable, the time before finding the reward). We looked at the interaction *conditioning odorant* × *trials* to detect possible differences in ants’ responses depending on the odorant used.

#### 2.4.2. Memory tests

We analysed the effects of four independent variables: *stimulus* (factor with two levels, CS and N), the time elapsed since conditioning (factor with four levels, 1, 24, 72 and 168 h, called *elapsed time*), the number of conditioning trials (factor with two levels, one or six conditioning trials, called *conditioning groups*) and the *conditioning odorant* on the dependent variable *time* (continuous variable, the time spent in the vicinity of a stimulus). We used post hoc tests to see whether the time spent in the CS or N area varies according to the time elapsed since conditioning. We tested whether ants spent more time in the CS or N area in function of the elapsed time since the conditioning.

For the memory tests performed in the pharmacological experiment (CHX), we tested if ants spent more time in the CS or N area 1 or 72 h after the conditioning trial as a function of treatment (CHX or control).

#### 2.4.3. Extinction

We analysed the effects of four independent variables: *stimulus*, the number of conditioning trials (factor with 3 levels, 1, 3 or 6 conditioning trials, called *conditioning groups*), the number of extinction trials (continuous variable from 1 to 12, called *extinction trials*) and the *conditioning odorant* on the dependent variable *time* (continuous variable, the time spent in the vicinity of a stimulus). At each extinction (or spontaneous recovery) trial, we tested if ants spent more time in the CS or in the N area.

## 3. Results

### 3.1. Acquisition

During the acquisition phase, the time spent by ants to find the reward decreased significantly over trials (LMM: *F* = 72.45, d.f. = 5, *p* < 0.001) (electronic supplementary material, figure S2). The observed decrease in the time to find the reward suggests that ants have associated the odorant (CS) with the reward (US). To validate that learning occurred, ants underwent memory tests without reward.

### 3.2. Memory tests

**Figure 2.**
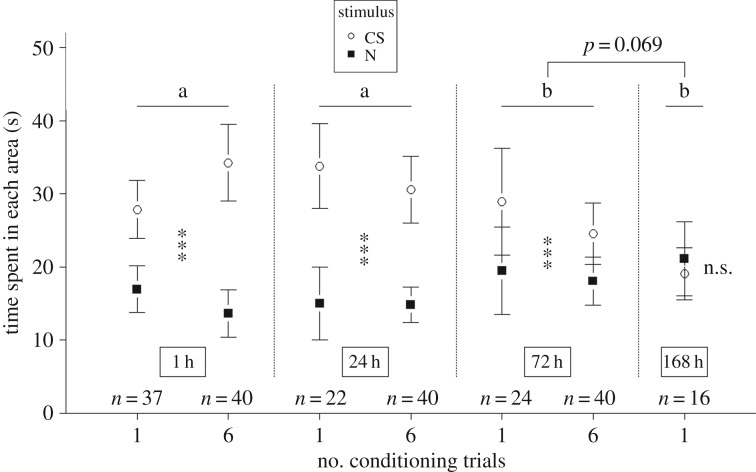
Time spent by individual ants in each area during the memory tests (white circle: CS area; black square: N area). Memory tests were performed 1, 24, 72 or 168 h after the end of the conditioning. Ants underwent one or six conditioning trials. Circles and squares represent the mean and error bars are confidence intervals (95%). Significant differences within a group are noted with asterisks (***p < 0.001; n.s.: *p* ≥ 0.05). Significant differences between groups (1, 24, 72 and 168 h) are noted with letters.

The number of conditioning trials (one or six) did not influence the performance (time spent near the CS) of the ants in the memory tests, as indicated by the non-significant interaction *stimulus* × *conditioning groups* (*F* = 0.52, d.f. = 1, *p* > 0.05). However, the time elapsed since conditioning had a significant effect on ants' behaviour (figure 2), as indicated by the significant interaction *stimulus* × *elapsed time* (*F* = 7.89, d.f. = 3, *p* < 0.001). Post hoc tests showed that the time spent in the stimuli areas was not significantly different when memory tests were performed 1 or 24 h after conditioning (*stimulus* × *elapsed time*, *p* > 0.1). A tendency was observed between 72 and 168 h (*p* = 0.069): ants performed better, i.e. spent more time in the CS area, at 72 h than at 168 h.

Instead, there was a significant difference in the ants’ performance between 1 and 72 h, with ants performing better at 1 h than at 72 h (*stimulus* × *elapsed time*, *p* < 0.05), between 24 h and 72 h (better performance at 24 h, *p* < 0.05), between 1 and 168 h (better performance at 1 h, *p* < 0.001) and between 24 and 168 h (better performance at 24 h, *p* < 0.001).

Finally, we observed that ants spent significantly more time in the CS than in the N area for the memory tests performed at 1, 24 and 72 h (for the three cases, 65.6 > *F* > 13.1, d.f. = 1, *p* < 0.001), while no significant difference was found at 168 h (*F* = 0.49, d.f. = 1, *p* > 0.1, figure 2), indicating that the ants do not prefer the CS to the N anymore. These results show that ants are capable of forming an appetitive associative memory that lasts for at least 3 days even after a single conditioning trial.

### 3.3. Pharmacological treatment

**Figure 3.**
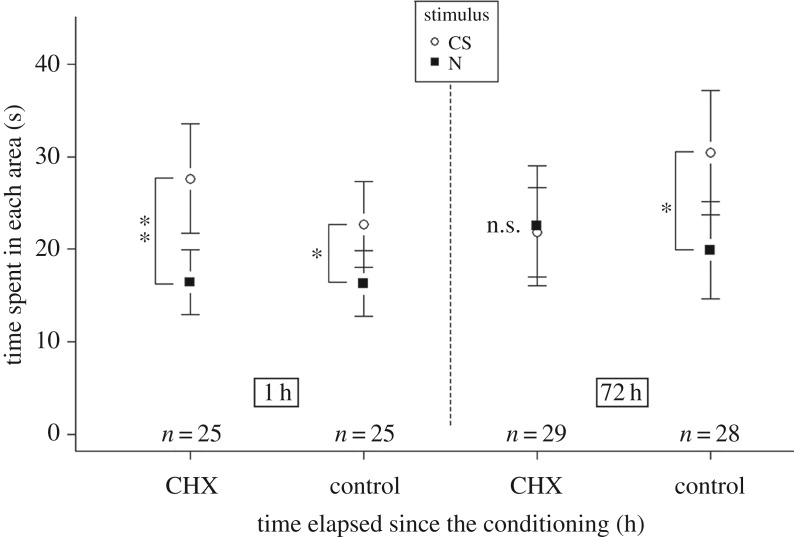
Time spent by individual ants in each area during memory tests. Ants received a drop of sugar solution 3 h before a single conditioning trial (control). In the experimental group (CHX), ants received 1 µg of CHX with the sugar solution. Memory tests were performed 1 h (left) or 72 h (right) after conditioning. Significant differences within a group are noted with asterisks (**p < 0.01; *p < 0.05; n.s.: *p* ≥ 0.05). Circles and squares represent the mean and error bars are confidence intervals (95%).

After a single conditioning trial, the olfactory memory of *F. fusca* appears to be very strong. We hypothesized that, even with such short training, ants built a LTM. To investigate this, we tested the susceptibility of this single-trial memory to a protein synthesis inhibitor (CHX). During these memory tests, control ants (sham-treated) spent more time in the CS area than in the N area, no matter whether the test was performed 1 h (*F* = 4.16, d.f. = 1, *p* < 0.05) or 72 h after conditioning (*F* = 6.03, d.f. = 1, *p* < 0.05), thus showing intact memory retention after 3 days. CHX-treated ants, however, displayed a preference for the CS area at 1 h (*F* = 8.62, d.f. = 1, *p* < 0.01) but not at 72 h after conditioning (*F* = 0.02, d.f. = 1, *p* > 0.05) (figure 3). This show that ants establish an appetitive olfactory LTM [37] after a single conditioning trial.

### 3.4. Extinction

#### 3.4.1. After six conditioning trials

**Figure 4.**
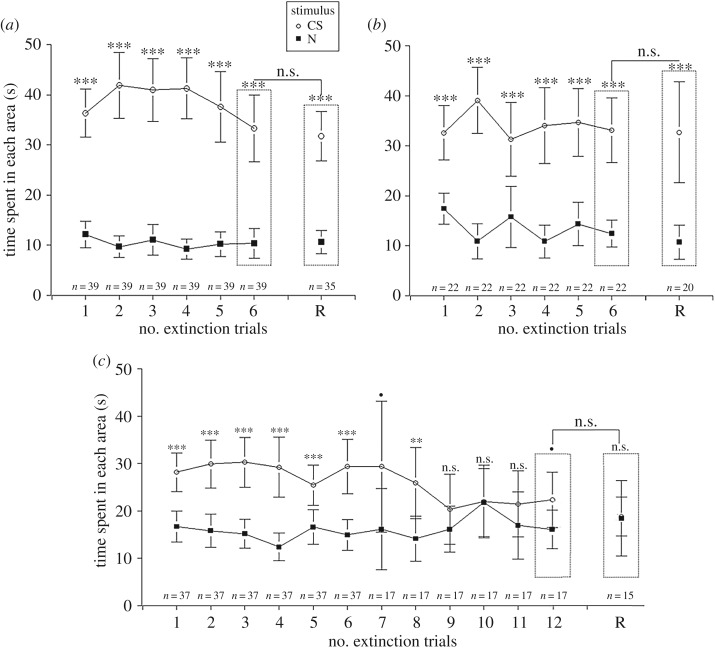
Time spent by ants in the CS and the N area during an extinction protocol that began 1 h after the last conditioning trial. *Y*-axis: time spent in the CS (white circles) and the N area (black squares). *X*-axis: number of extinction trials (6–12). The test for spontaneous recovery (24 h after the last extinction trial) is represented by the letter ‘R’. Different panels represent different groups of ants: (*a*) ants that underwent six conditioning trials and six extinction trials (*n* = 39), (*b*) ants with three conditioning trials and six extinction trials (*n* = 22) (*c*) and ants with one conditioning trial and six (*n* = 20) and 12 extinction trials (*n* = 17). Significant differences within extinction trials or between the last extinction trial and the test of spontaneous recovery are noted with asterisks (***p < 0.001; **p < 0.01; *p < 0.05; n.s.: *p* ≥ 0.0.5). Circles and squares represent the mean and error bars are confidence intervals (95%).

Ants did not show any significant extinction in the course of the six-test procedure (figure 4*a*). Indeed, the ants' performance was stable over time, as indicated by the non-significant *stimulus* × *extinction trials* interaction (*F* = 1.40, d.f. = 5, *p* > 0.05). When comparing the ant performance in the last extinction trial and in the spontaneous recovery test, no significant difference was found (*F* = 0.10, d.f. = 1, *p* > 0.05) (figure 4*a*). In all the extinction trials and in the spontaneous recovery test, ants spent more time in the CS than in the N area (142.7 > *F* > 50.54, d.f. = 1, *p* < 0.001). Therefore, with six conditioning trials, ants showed high resistance to extinction.

#### 3.4.2. After three conditioning trials

Given that extinction did not occur after six conditioning trials, we decided to subject ants to only three conditioning trials before the six extinction trials. Again, we found that the *stimulus* × *extinction trials* interaction was non-significant, indicating that ants’ performance was stable during the extinction procedure (*F* = 2.14, d.f. = 5, *p* > 0.05) (figure 4*b*). When comparing the last extinction trial with the spontaneous recovery test, we did not find any significant difference (*F* = 0.01, d.f. = 1, *p* > 0.05). In all the extinction trials and in the spontaneous recovery test, ants spent more time in the CS than in the N area (71.7 > *F* > 15.15, d.f. = 1, *p* < 0.001). Here, again, ants show very high resistance to extinction.

#### 3.4.3. After one conditioning trial and up to 12 extinction trials

Even with three conditioning trials, no extinction was found after six extinction trials. We then further reduced training strength and subjected the ants to a single conditioning trial before the extinction procedure. If we look at the first six extinction trials, the ants' performance did not decrease along the trials (figure 4*c*) as indicated by the non-significant interaction *stimulus* × *extinction trials* (*F* = 0.67, d.f. = 5, *p* > 0.1). For these ants, which underwent one conditioning trial and six extinction trials, there is no significant difference between the sixth extinction trial and the test of spontaneous recovery (*F* = 0.17, d.f. = 1, *p* > 0.1). For the first six trials and the test of spontaneous recovery, ants spent significantly more time in the CS than in the N area (28.38 > *F* > 12.62, d.f. = 1, *p* < 0.001) (figure 4*c*). Some of the ants that received only one conditioning trial underwent six additional extinction trials. Overall, the ants’ performances (i.e. the time spent near the CS) decreased along the 12 tests, indicating extinction (figure 4*c*), as shown by the significant *stimulus* × *extinction trials* interaction (*F* = 1.83, d.f. = 11, *p* < 0.05). To detect at which trial extinction started, we tested if ants spent more time in the CS or N area during the last six extinction trials. For the seventh trial, a tendency is observed (*F* = 3.69, d.f. = 1, *p* = 0.063), for the eighth trial, ants spent more time in the CS area (*F* = 8.74, d.f. = 1, *p* < 0.01) but at the 9th, 10th and 11th trials, ants did not show any significant preference for one of the stimuli (0.88 > *F* > 0.001, d.f. = 1, *p* > 0.1). For the last extinction trial, a tendency was observed (*F* = 4.14, d.f. = 1, *p* = 0.051). Finally, no significant difference was found between ants' performances in the 12th extinction trial and in the spontaneous recovery test (*F* = 0.75, d.f. = 1, *p* > 0.05). During this spontaneous recovery test, ants did not spend more time in the CS area compared to the N area (*F* = 0.26, d.f. = 1, *p* > 0.1), indicating that spontaneous recovery did not occur within 24 h.

## 4. Discussion

By using a simple conditioning paradigm, our study shows that *F. fusca* ants are very efficient in individual olfactory learning, and reveals unconventional characteristics in their learning ability. These ants learn an odour-reward association very quickly (within a single trial) and thereby build a highly stable memory form (genuine LTM, dependent on protein synthesis), which was undescribed in ants. Moreover, the established odour-reward association is highly resistant to contradictory information, being subject to extinction only after many unrewarded trials.

### 4.1. Formation of a long-term memory after one conditioning trial

During conditioning with three and six conditioning trials, ants showed fast acquisition: the time to find the reward rapidly decreased after the first conditioning trial, suggesting that memory is already formed after a single conditioning trial. When ants that underwent one or six conditioning trials were tested with an unrewarded memory test (1, 24 or 72 h after conditioning), no significant differences in ants’ performances were found between the one conditioning trial group and the six conditioning trials group. Typically, in insects as diverse as fruit flies, crickets and honeybees, a single conditioning trial results in the formation of a short-term memory (lasting 1–24 h), whereas several conditioning trials are needed to form an LTM (lasting more than 24 h) [33,38,39]. In ants, a recent study showed that one training trial is sufficient for olfactory learning, but if this leads to LTM was not known [23]. To test whether the memory formed after a single conditioning trial is genuine LTM, we treated ants with a protein synthesis inhibitor (cycloheximide) before conditioning and we observed that, 72 h later, treated ants could not retrieve any memory. This confirms that the memory formed by *F. fusca* ants after a single conditioning trial is true LTM [11,22,40]. The formation of an LTM after one single appetitive conditioning trial is not common in insects, and has been found until now only in the fruit fly, *Drosophila melanogaster* [11,41].

To investigate the limit of this memory, we tested ants one week after this one single conditioning trial and no trace of memory was found. We did not perform this test at one week for ants that underwent six conditioning trials, but we assume that they would behave similarly since their performances were not different from ants that underwent one conditioning trial when tested after 1, 24 and 72 h.

Our study is original in that it shows both single-trial olfactory learning and the formation of a highly stable memory form after this single learning. Single-trial visual learning has been shown in individual foragers of desert ants, for example, *Melophorus bagoti*, but it is unclear whether this short training leads to LTM [17]. In the case of olfactory learning, previous studies found that ants can learn rapidly or retain memories for a long time, but both abilities were rarely found together. Moreover, these studies were performed at the colony level (not at the individual level) and/or involved very young individuals in an imprinting context. Workers of the desert ant *Cataglyphis fortis* can collectively learn to associate one odorant with food after one trial, and about half of the ants remember this association for up to 26 days afterwards [42]. Leaf-cutting ants, *Atta colombica*, feed their symbiotic fungus with freshly collected leaves. Field colonies learn to avoid plants that are dangerous for their fungus (e.g. experimentally treated with fungicide) and show robust memory for plant unsuitability lasting up to 18 weeks [43].

Young workers of *Formica polyctena* that were reared just after emergence with cocoons of an alien species for 15 days will take care of cocoons of this species when encountering them six months later, while they will eat conspecific cocoons that they never encountered [44]. This is an imprinting-like phenomenon occurring during a critical period after emergence and that has been shown in ants several times (review in [45]). Imprinting-like phenomena also occur with environmental odorants. If young ants are reared from eclosion in a nest with a specific plant odorant (i.e. thyme), and are then kept in a nest without odorant, they will prefer a nest with the odour experienced during their young age when given the choice [46]. Young individuals can possibly form long-term olfactory memories that persist for weeks or even months. Indeed, high retention abilities were found when training cricket nymphs to discriminate an odorant associated with water or saline solution [47], and in very young mice trained to associate an odorant with milk [48], especially after a period of deprivation from these resources. In the present case, we documented ants' adult learning abilities at the individual level and show that they build long-lasting olfactory memories within a single rewarded trial.

### 4.2. *Formica fusca* ants are particularly resistant to extinction

The second part of our study involved testing resistance to extinction. Based on the social insect literature, we expected to observe a rapid decrease in performances along extinction trials. In honeybees, using the proboscis extension response conditioning paradigm, 80% of the bees show a conditioned response after six conditioning trials. After two extinction trials, 70% of these bees display the conditioned response, and after five extinction trials, 60% still respond. However, with a single conditioning trial, only 10.7% of bees show the conditioned response after five extinction trials [15]. In *Myrmica* ants tested at the colony level, two extinction trials are needed to extinguish an olfactory association established with a 12 conditioning trials procedure [49]. In our first extinction protocol, with ants that underwent six conditioning trials and six unrewarded extinction trials, no extinction was observed. This result in itself is surprising and was replicated with ants that underwent three or only one single conditioning trial. We could observe extinction only in ants that underwent a single conditioning trial and more than six extinction trials, demonstrating an exceptionally high resistance to extinction in these ants. During extinction, the memory formed after conditioning (CS–US association) is not erased, but a new learning usually takes place (CS–no US association) [32]. The behaviour of the individual will reflect the relative strength of each memory. With more extinction trials, the CS–no US association will become stronger than the CS–US memory and individuals will stop responding to the CS. As the CS–US memory is not erased, if we test the behaviour of an individual at a later time after the end of the extinction protocol, positive response to the CS may increase again. This phenomenon, called spontaneous recovery [50], usually corresponds to a decay of the CS–no US memory. Here, we did not observe any spontaneous recovery when extinction took place (i.e. one conditioning trial and 12 extinction trials). This absence of spontaneous recovery could be due to the time elapsed between the end of the extinction trials and spontaneous recovery test, which may have been too short for the decay of the CS–no US association. In honeybees, spontaneous recovery usually appears within a few hours (1 h for [18], 35 min for [51]). In any case, the lack of spontaneous recovery in these ants confirms the stability of the associations they form: just like their CS–US association is highly resistant to time and extinction, their CS–no US association also appears to be highly resistant to time.

Why do ants show such fast learning and high resistance to extinction? In ants, as in other social insects, individuals are usually specialized in a particular task according to their age. Young workers will avoid threats, stay in the nest and take care of the brood, whereas old workers will go out foraging, and therefore be exposed to biotic and abiotic threats [52]. In undisturbed natural colonies, as workers keep the same job for weeks or even months, it is not relevant to learn rapidly and to be resistant to extinction. However, if a category of workers suddenly decreases in number (e.g. due to predation or raids from slave-making ants, of which *F. fusca* is a common host species [53]), task switching may occur and workers with the ability to learn quickly will be very advantageous for the colony. Being resistant to extinction is an advantage when the environment is extremely stochastic and workers need to switch often from a current task to another, previously learned, task. In *F. fusca*, there is no clear specialization of individuals working outside the nest, which may engage in different tasks such as foraging, scouting and guarding [54]. An ant could act as forager one day, guard another day and then forager again. In this scenario, learning quickly and building strong memories of a previously learned task (i.e. being resistant to extinction) is advantageous because it allows ants to exploit optimally their environment without spending time to learn again.

After documenting the unconventional olfactory learning and memory abilities of *F. fusca*, we are left wondering if this species is really exceptional among ants, and more generally among social insects. While visual learning is well documented in ants, especially in the context of navigation [18], data on individual olfactory learning and memory abilities in ants are relatively scarce and future comparative studies taking into account the ant phylogeny may be useful to provide answers to this question. Furthermore, given that the insect brain shares many similarities in its architecture with the vertebrate brain [55], a better understanding of the neural mechanisms underlying such a stabilized memory and resistance to extinction might help improving treatment of maladaptive behaviours.

## Supplementary Material

Supplementary methods

Reviewer comments

## Supplementary Material

Figure and tables

## Supplementary Material

original data

## Data accessibility

Original data are accessible in electronic supplementary material, together with supplementary methods and results, tables S1–S5 and figures S1 and S2.
