# Supplementary material for: Ants learn fast and do not forget: associative olfactory learning, memory and extinction in Formica fusca
Source: R Soc Open Sci. 2019 Jun 19;6(6):190778. doi: 10.1098/rsos.190778 (PMC6599790; doi:10.1098/rsos.190778)
Supplement: Supplementary methods [file rsos190778supp1.pdf]

Electronic Supplementary Information

Ants learn fast and do not forget: olfactory associative learning, memory and extinction in *Formica fusca*

Baptiste Piqueret, Jean-Christophe Sandoz, Patrizia d'Ettorre

Methods

Odorant stimuli

Due to their low molecular weight, hexanal and 1-octanol are volatile at ambient temperature. We tested whether ants might show a spontaneous preference for these odorants, but this is not the case. When individual ants were presented in a circular arena (Fig. 1A) with hexanal and 1-octanol at two opposite sides, the ants did not show any preference (Linear Mixed Model: $F = 0.10$, df = 1, $p > 0.1$, N = 10). The two odorants are clearly different from a chemical point of view and were shown to induce low behavioural generalisation in honey bees (1). Therefore, they are expected to be perceived as clearly different odorants by the ants (2).

Pharmacological treatment

To exclude a possible effect of CHX on the ants' health, we carried out a control experiment in which ants received CHX (or only sugar solution) 72h - instead of 3h - before a single conditioning trial. We then performed a memory test 1h after conditioning. No difference was observed between treated (CHX) and control ants, demonstrating that CHX does not affect the physical conditions of ants (Linear Mixed Model: *stimulus* × *treatment:* $F = 2.20$, df = 1, $p > 0.1$, N = 17 treated and N = 16 control ants, table S4).

Detailed statistical methods

Data were analysed using R software (v 3.5.2, R Core Team, 2018 (3)). Significance was fixed at $\alpha = 5\%$. Data were transformed with neperian logarithm or square root, depending on which transformation was the best to approach normality. Homogeneity of variance was checked for all the full models. All data were analysed using Linear Mixed Models (LMM, package "lme4", (4)). To allow repeated measurements and adjust for colony origin, individual identity was coded as random factor nested into colony origin. *F* and *p*-values from the LMM were calculated using a Wald-test with Satterthwaite's correction ("car" package, (5)). Post-hoc differences were observed by using LMMs with reduced dataset and the alpha level was adjusted using Holm-Bonferroni Correction (6).

Acquisition

We analysed the effects of two independent (predictor) variables: '*conditioning odorant'* (factor with 2 levels, hexanal or 1-octanol) and the number of conditioning trials (continuous variable up to 6, named '*trials'*) on the dependant variable '*time*' (continuous variable, the time before finding the reward). We looked at the interaction *conditioning odorant* × *trials* to detect possible differences in ants' responses depending on the odorant used. The conditioning odorant did not influence the acquisition dynamics (LMM: $F = 0.91$, df = 5, $p > 0.05$). We then ran a simplified model to test the effect of the conditioning trials on acquisition. We also ran a post hoc analysis to compare each conditioning trial with the others; all the 15 possible comparisons (trial 1 *vs* trial 2, trial 1 *vs* trial 3…) were tested and the Holm-Bonferroni correction was applied (Table S1).

i) Memory tests

For the memory tests, we analysed the effects of four independent variables: 'stimulus' (factor with 2 levels, CS and N), the time elapsed since conditioning (factor with 4 levels, 1h,

24h, 72h and 168h, called '*elapsed time*'), the number of conditioning trials (factor with 2 levels, 1 or 6 conditioning trials, called '*conditioning groups*') and the '*conditioning odorant*' (factor with 2 levels, hexanal or 1-octanol) on the dependant variable '*time*' (continuous variable, the time spent in the vicinity of a stimulus). We looked at the triple interaction between *stimulus* × *elapsed time* × *conditioning group,* which was not significant (LMM: F = 1.77, df = 4, p > 0.05). A simplified model was run where we looked at the interactions between *stimulus* × *elapsed time* and *stimulus* × *conditioning groups.* We then used post-hoc tests to see whether the time spent in the CS or N area varies according to the time elapsed since conditioning. We finally tested whether ants spent more time in the CS or N area in function of the elapsed time since the conditioning.

For the memory tests performed in the pharmacological experiment (CHX), we tested if ants spent more time in the CS or N area 1h or 72h after the conditioning trial as a function of treatment (CHX or control).

ii) *Extinction*

We analysed the effects of four independent variables: 'stimulus' (factor with 2 levels, CS and N), the number of conditioning trials (factor with 3 levels, 1, 3 or 6 conditioning trials, called '*conditioning groups*'), the number of extinction trials (continuous variable from 1 to 12, called '*extinction trials')* and the '*conditioning odorant*' (factor with 2 levels, hexanal or 1-octanol) on the dependant variable '*time*' (continuous variable, the time spent in the vicinity of a stimulus). In the full model, we observed that the interaction *stimulus* × *conditioning group* was significant (LMM: F = 35.36, df = 2, p < 0.001), so we ran different models for the different conditioning groups (1, 3 or 6 conditioning trials). For the simplified model, we looked at the interaction *stimulus* × *extinction trials* to detect extinction.

To test for spontaneous recovery, we ran a model with a subset of data consisting only in the last extinction trial and the test of spontaneous recovery and looked at the *stimulus* × *extinction trials* interaction. Finally, at each extinction (or spontaneous recovery) trial, we tested if ants spent more time in the CS or in the N area.

References

1. Guerrieri F, Schubert M, Sandoz JC, Giurfa M. Perceptual and neural olfactory similarity in honeybees. PLoS Biol. 2005;3(4):0718–32.
2. Dupuy F, Josens R, Giurfa M, Sandoz JC. Calcium imaging in the ant Camponotus fellah reveals a conserved odour-similarity space in insects and mammals. BMC Neurosci. 2010;11.
3. R Core Team. R: A Language and Environment for Statistical Computing [Internet]. Vienna, Austria: R Foundation for Statistical Computing; 2018. Available from: https://www.r-project.org/
4. Bates D, Mächler M, Bolker B, Walker S. Fitting Linear Mixed-Effects Models Using lme4. J Stat Softw [Internet]. 2015;67(1):1–48. Available from: http://www.jstatsoft.org/v67/i01/
5. Fox J, Weisberg S. An {R} Companion to Applied Regression. Sage Publications. Thousand Oaks {CA}: Sage; 2011. p. 2–3.
6. Holm S. A Simple Sequentially Rejective Multiple Test Procedure. Scand J Stat [Internet]. 1979;6(2):65–70. Available from: https://www.ime.usp.br/~abe/lista/pdf4R8xPVzCnX.pdf
