# Supplementary material for: Ants learn fast and do not forget: associative olfactory learning, memory and extinction in Formica fusca
Source: R Soc Open Sci. 2019 Jun 19;6(6):190778. doi: 10.1098/rsos.190778 (PMC6599790; doi:10.1098/rsos.190778)
Supplement: Figure and tables [file rsos190778supp2.pdf]

Electronic Supplementary Information

# Ants learn fast and do not forget: olfactory associative learning, memory and extinction in *Formica fusca*

Baptiste Piqueret, Jean-Christophe Sandoz, Patrizia d'Ettorre

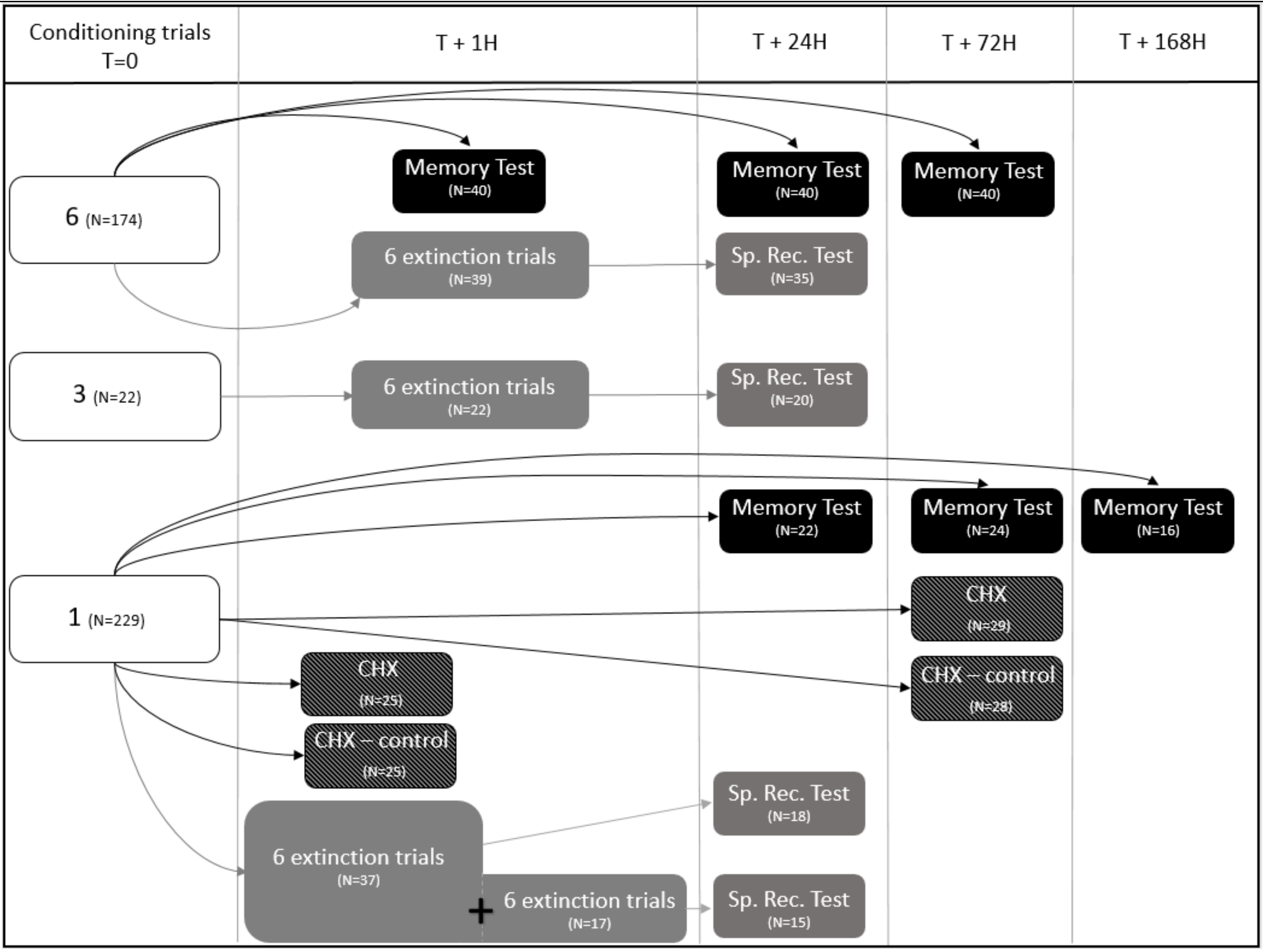


**Figure S1**: Overview of the conducted experiments. The left column shows the number of conditioning trials ants were subjected to (white box). Three different experiments were performed. Independent groups of ants underwent a memory test (black boxes), an extinction protocol (grey boxes) or were treated with CHX before performing a memory test (black striped boxes). Tests were conducted 1 h, 24 h, 72 h or 168 h after the end of conditioning. For the ants that underwent an extinction protocol, a test of spontaneous recovery (Grey boxes: Sp. Rec. Test) was performed 24h after the last extinction trial. N= number of ants.

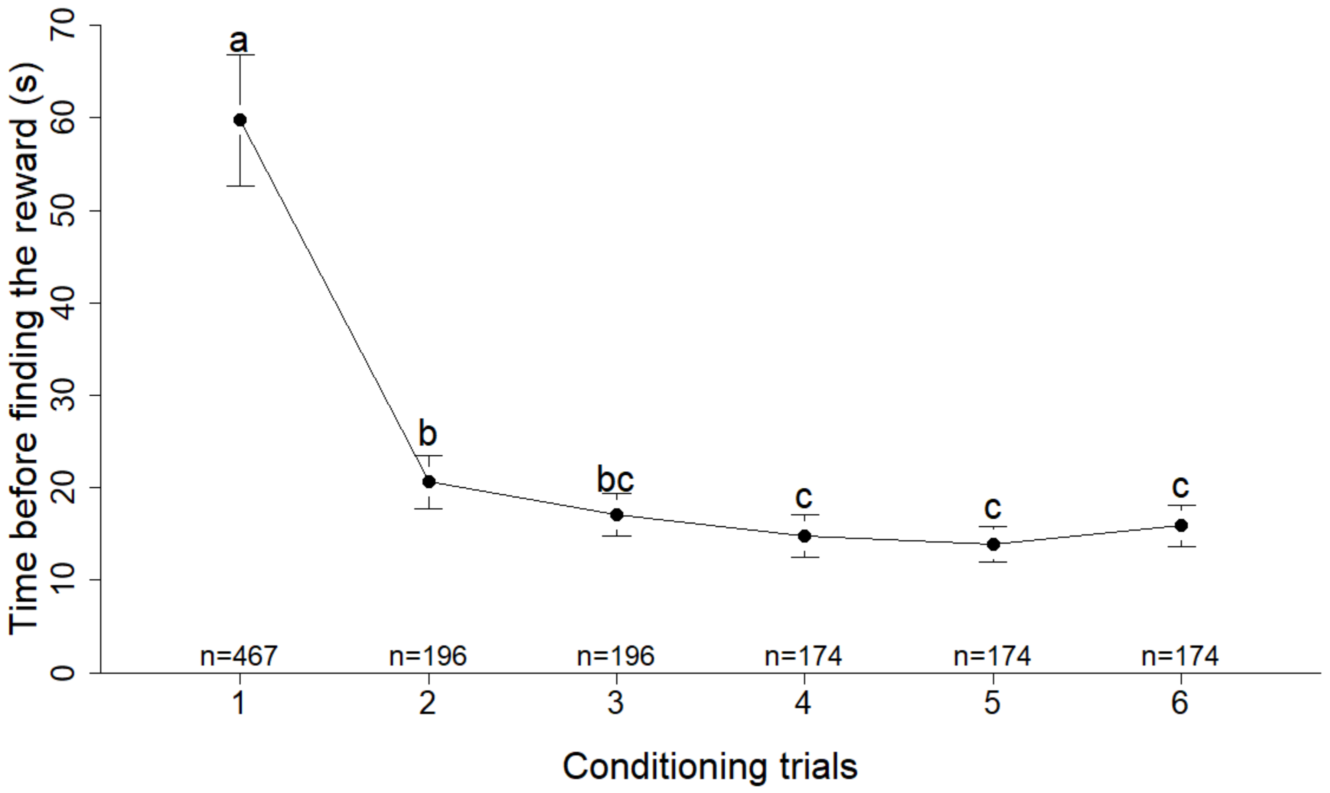


**Figure S2**: Time (mean + 95% confidence interval) spent by the ants to find the reward along the conditioning trials (N = 174 for six trials, N = 22 for three trials, N = 271 for one trial). Letters indicate difference between trials after Holm-Bonferroni correction. In particular, the first trial was different from all the other trials, in which the time to find the reward was significantly shorter (post-hoc, $p$ < 0.001 for all comparison, after Holm-Bonferroni correction). The second and third trials were not significantly different ($p$ > 0.05); however, the second trial was different from all the other trials ($p$ < 0.05 in all cases). There was no difference from the third trial to the sixth (p > 0.05 in all cases).

**Table S1|** Result of the different model used during the analyses of the ant conditioning (Fig S3): Time was always the dependent variable. Significant effects are given in bold.

| Focus on the factor(s) | Df | F value | p value |
|---|---|---|---|
| **Conditioning odorant × trials × Conditioning groups** | 2 | 0.9271 | 0.396 |
| | | | |
| **Conditioning odorant × trials** | 5 | 0.9101 | 0.473 |
| | | | |
| Trials | 5 | 72.4502 | **<0.001** |
| Trials (1 vs 2) | | | **<0.001** |
| Trials (1 vs 3) | | | **<0.001** |
| Trials (1 vs 4) | | | **<0.001** |
| Trials (1 vs 5) | | | **<0.001** |
| Trials (1 vs 6) | | | **<0.001** |
| Trials (2 vs 3) | | | 0.287 |
| Trials (2 vs 4) | | | **0.007** |
| Trials (2 vs 5) | | | **<0.001** |
| Trials (2 vs 6) | | | **0.048** |
| Trials (3 vs 4) | | | 0.685 |
| Trials (3 vs 5) | | | 0.270 |
| Trials (3 vs 6) | | | 1.000 |
| Trials (4 vs 5) | | | 1.000 |
| Trials (4 vs 6) | | | 1.000 |
| Trials (5 vs 6) | | | 0.680 |

**Table S2 |** Results of the model used during the analyses of the memory tests (Fig 3): Time was the dependent variable; *p*-value are adjusted with Bonferronni-Holm correction when needed. Significant effects are given in bold. Tendency effects are underlined.

| Interaction or factor analysed | Post-hoc | Df | F value | p value |
|---|---|---|---|---|
| elapsed time × conditioning groups × stimulus | | 4 | 1.7707 | 0.133 |
| | | | | |
| conditioning groups × stimulus | | 1 | 0.5172 | 0.472 |
| stimulus × elapsed time | | 3 | 7.8924 | **<0.001** |
| | | | | |
| stimulus × elapsed time | 1h/24h | 1 | | 0.716 |
| stimulus × elapsed time | 1h/72h | 1 | | **0.022** |
| stimulus × elapsed time | 1h/168h | 1 | | **<0.001** |
| stimulus × elapsed time | 24h/72 | 1 | | **0.012** |
| stimulus × elapsed time | 24h/168h | 1 | | **<0.001** |
| stimulus × elapsed time | 72h/168h | 1 | | 0.068 |
| Stimulus | 1h | 1 | 62.997 | **<0.001** |
| Stimulus | 24h | 1 | 65.597 | **<0.001** |
| Stimulus | 72h | 1 | 13.141 | **<0.001** |
| Stimulus | 168h | 1 | 0.488 | 0.495 |

**Table S3 |** Results of the model used during the analyses of the effect of the cycloheximide (CHX, given 3 hours before the conditioning trial) on memory retention (Fig 4): Time was the dependent variable. Significant effects are given in bold. Tendency effects are underlined.

| Interaction or factor analysed | Post-hoc | Was CHX given? | Df | F value | p value |
|---|---|---|---|---|---|
| Treatment × Stimulus | | | | 0.783 | 0.378 |
| Stimulus | 1h | No | 1 | 4.167 | **0.046** |
| Stimulus | 1h | Yes | 1 | 8.627 | **0.005** |
| Treatment × Stimulus | | | | 2.907 | 0.091 |
| Stimulus | 72h | No | 1 | 6.039 | **0.017** |
| Stimulus | 72h | Yes | 1 | 0.025 | 0.872 |

**Table S4 |** Results of the model used during the analyses of the effect of the cycloheximide (CHX, given 72 hours before the conditioning trial) on memory retention: Time was the dependent variable. Significant effects are given in bold.

| Interaction or factor analysed | Post-hoc | Df | F value | p value |
|---|---|---|---|---|
| Treatment × Stimulus | | 1 | 2.204 | 0.143 |
| Stimulus | CHX | 1 | 24.468 | **<0.001** |
| Stimulus | Control | 1 | 12.590 | **0.001** |

**Table S5 |** Results of the model used during the analyses of the extinction procedure (Fig 5): Time was the dependent variable. Significant effects are given in bold. Tendency effects are underlined.

| Interaction or factor analysed | Post-hoc | Df | F value | p value |
|---|---|---|---|---|
| stimulus × conditioning group | | 2 | 35.621 | **<0.001** |
| | | ***Six conditioning trials*** | | |
| stimulus × extinction trials | | 5 | 1.397 | 0.224 |
| Stimulus | Trial 1 | 1 | 106.180 | **<0.001** |
| Stimulus | Trial 2 | 1 | 127.940 | **<0.001** |
| Stimulus | Trial 3 | 1 | 102.750 | **<0.001** |
| Stimulus | Trial 4 | 1 | 142.700 | **<0.001** |
| Stimulus | Trial 5 | 1 | 74.979 | **<0.001** |
| Stimulus | Trial 6 | 1 | 50.542 | **<0.001** |
| Stimulus | Trial Recuperation | 1 | 66.753 | **<0.001** |
| stimulus × extinction trials | Trial 6/ Recuperation | 1 | 0.104 | 0.746 |
| | | ***Three conditioning trials*** | | |
| stimulus × extinction trials | | 5 | 2.140 | <u>0.061</u> |
| Stimulus | Trial 1 | 1 | 29.489 | **<0.001** |
| Stimulus | Trial 2 | 1 | 71.702 | **<0.001** |
| Stimulus | Trial 3 | 1 | 15.145 | **<0.001** |
| Stimulus | Trial 4 | 1 | 35.551 | **<0.001** |
| Stimulus | Trial 5 | 1 | 28.973 | **<0.001** |
| Stimulus | Trial 6 | 1 | 49.574 | **<0.001** |
| Stimulus | Trial Recovery | 1 | 19.645 | **<0.001** |
| stimulus × extinction trials | Trial 6/Recovery | 1 | 0.008 | 0.926 |
| | | ***One conditioning trial*** | | |
| Stimulus × extinction trials | | 11 | 1.825 | **0.046** |
| Stimulus | Trial 1 | 1 | 16.689 | **<0.001** |
| Stimulus | Trial 2 | 1 | 23.354 | **<0.001** |
| Stimulus | Trial 3 | 1 | 28.386 | **<0.001** |
| Stimulus | Trial 4 | 1 | 27.947 | **<0.001** |
| Stimulus | Trial 5 | 1 | 12.617 | **<0.001** |
| Stimulus | Trial 6 | 1 | 22.053 | **<0.001** |
| Stimulus | Trial 7 | 1 | 3.687 | <u>0.063</u> |
| Stimulus | Trial 8 | 1 | 8.742 | **0.005** |
| Stimulus | Trial 9 | 1 | 0.883 | 0.354 |
| Stimulus | Trial 10 | 1 | 0.001 | 0.975 |
| Stimulus | Trial 11 | 1 | 0.874 | 0.356 |
| Stimulus | Trial 12 | 1 | 4.139 | <u>0.051</u> |
| Stimulus | Trial Recovery 6 | 1 | 22.597 | **<0.001** |
| Stimulus | Trial Recovery 12 | 1 | 0.262 | 0.612 |
| Stimulus × extinction trials | Trial 6/ Recovery 6 | 1 | 0.177 | 0.675 |
| Stimulus × extinction trials | Trial 12/ Recovery 12 | 1 | 0.749 | 0.390 |
