# Supplementary material for: Ants learn fast and do not forget: associative olfactory learning, memory and extinction in Formica fusca
Source: R Soc Open Sci. 2019 Jun 19;6(6):190778. doi: 10.1098/rsos.190778 (PMC6599790; doi:10.1098/rsos.190778)
Supplement: Reviewer comments [file rsos190778_review_history.pdf]

THE ROYAL SOCIETY
PUBLISHING

ROYAL SOCIETY
OPEN SCIENCE

Ants learn fast and do not forget: associative olfactory learning, memory and extinction in *Formica fusca*

Baptiste Piqueret, Jean-Christophe Sandoz and Patrizia d'Ettorre

Article citation details
R. Soc. open sci. **6**: 190778.
http://dx.doi.org/10.1098/rsos.190778

Review timeline
Original submission: 25 April 2019
Final acceptance: 21 May 2019

Note: Reports are unedited and appear as submitted by the referee. The review history appears in chronological order.

Note: This manuscript was transferred from another Royal Society journal with peer review.

Review History

Decision letter (RSOS-190778.R0)

21-May-2019

Dear Mr Piqueret:

It is a pleasure to accept your manuscript entitled "Ants learn fast and do not forget: associative olfactory learning, memory and extinction in *Formica fusca*" in its current form for publication in Royal Society Open Science. The comments of the reviewer(s) who reviewed your manuscript are included at the foot of this letter.

Thank you for your fine contribution. On behalf of the Editors of Royal Society Open Science, we look forward to your continued contributions to the Journal.

Kind regards,
Andrew Dunn
Royal Society Open Science Editorial Office
Royal Society Open Science
openscience@royalsociety.org

Reports © 2019 The Reviewers; Decision Letters © 2019 The Reviewers and Editors; Responses © 2019 The Reviewers, Editors and Authors. Published by the Royal Society under the terms of the Creative Commons Attribution License http://creativecommons.org/licenses/by/4.0/, which permits unrestricted use, provided the original author and source are credited

on behalf of Dr Alecia Carter (Associate Editor) and Dr Kevin Padian (Subject Editor).

Follow Royal Society Publishing on Twitter: @RSocPublishing
Follow Royal Society Publishing on Facebook:
https://www.facebook.com/RoyalSocietyPublishing.FanPage/
Read Royal Society Publishing's blog: https://blogs.royalsociety.org/publishing/
